# FOREWORD Finding Balance in the Fight Against Gun Violence

**DOI:** 10.1017/jme.2023.37

**Published:** 2023

**Authors:** Michael R. Ulrich

**Affiliations:** 1: BOSTON UNIVERSITY, BOSTON, MA, USA

**Keywords:** Gun Violence, Second Amendment, Disparities, Intersectionality, Firearms Justice

## Abstract

The United States is distinct among high-income countries for its problem with gun violence, with Americans 25 times more likely to be killed by gun homicide than people in other high-income countries.^1^ Suicides make up a majority of annual gun deaths — though that gap is closing as homicides are on the rise — and the U.S. accounts for 35% of global firearm suicides despite making up only 4% of the world’s population.^2^ More concerning, gun deaths are only getting worse. In 2021, firearm fatalities approached 50,000, the highest we have seen in at least 40 years.^3^ The increase in homicides in conjunction with lower crime overall further suggests an problem specifically with guns.^4^ As devastating as these deaths are, it does not come close to encompassing the mass toll of America’s gun violence epidemic — a toll that disproportionately impacts people of color, with the Black community suffering at the highest rates. A broader and more accurate view of what constitutes gun violence must become a part of the national discourse if we are going to develop effective strategies to combat this crisis.^5^

The United States is distinct among high-income countries for its problem with gun violence, with Americans 25 times more likely to be killed by gun homicide than people in other high-income countries.^1^ Suicides make up a majority of annual gun deaths — though that gap is closing as homicides are on the rise — and the U.S. accounts for 35% of global firearm suicides despite making up only 4% of the world’s population.^2^ More concerning, gun deaths are only getting worse. In 2021, firearm fatalities approached 50,000, the highest we have seen in at least 40 years.^3^ The increase in homicides in conjunction with lower crime overall further suggests an problem specifically with guns.^4^ As devastating as these deaths are, it does not come close to encompassing the mass toll of America’s gun violence epidemic — a toll that disproportionately impacts people of color, with the Black community suffering at the highest rates. A broader and more accurate view of what constitutes gun violence must become a part of the national discourse if we are going to develop effective strategies to combat this crisis.^5^

Discussions of gun violence in the U.S. must include nonfatal shootings, which can create long-term difficulties for physical and mental health. Guns can also generate harm without a trigger ever being pulled. Firearms can be used for threats and intimidation, for example, to perpetuate sexual violence and psychological torment. Police shootings can generate anxiety, post-traumatic stress disorder, feelings of helplessness and fear. Gun violence from law enforcement may even be a contributor to elevated rates of preterm delivery and cardiovascular disease in Black women.^6^ Long-term effects on youth may not be fully understood for years, but it should be unacceptable that a majority of high school students report concerns that a shooting will take place in their school or community.^7^ Active shooter drills, which create their own trauma and fear, have become normalized.^8^ Meanwhile, the impact gun violence can have on the exercise of rights, freedoms, and liberties for the broader public, such as going to the park, movies, or grocery store, cannot be ignored.^9^

Amidst this crisis, the United States Supreme Court delivered a crushing blow to those seeking legal options to mitigate this growing threat to public health and safety. With devastating rates of gun violence as its backdrop, the Supreme Court issued its first substantive Second Amendment ruling since declaring in *District of Columbia v. Heller* that the Constitution protected an individual right to own and operate a handgun in the home for self-defense.^10^ In *New York State Rifle & Pistol Association, Inc. v. Bruen*, the Court decided to expand that right beyond the home and into the public sphere.^11^ In this case the Court struck down a 1913 New York law that required people seeking a permit to carry a firearm in public to demonstrate a specific need to do so.^12^ To satisfy the “proper cause” requirement the individual had to provide a justification beyond a general desire to have a gun for the theoretical possibility they may need to defend themselves.^13^ This form of public carry regulation — often referred to as a “may issue” licensing regime — was in a minority of jurisdictions, but those included some of the most populated cities and ultimately covered nearly 80 million people.^14^

Striking down laws that limit firearms in public is concerning for its potential to increase gun violence. The most current research has found a strong association between increased gun violence and lax public carry regimes — either “shall issue” laws that remove discretion from law enforcement to grant public carry permits or “constitutional carry” states that do not require a permit for public carry at all.^15^ Yet, the potential to exacerbate an existing and growing public health crisis seemed irrelevant to the Court in *Bruen*. In writing for the majority, Justice Clarence Thomas gave no mention of gun violence. Instead, the focus was solely on looking back to the past. The Court’s analysis of New York’s authority to limit firearms in public came down to whether enough jurisdictions treated guns the same way centuries ago. According to the Court in *Bruen*, Second Amendment rights can only be limited in the same manner they were in the era of musktets and bayonets.^16^ And it is this histori-cal analytical framework used in *Bruen* that creates an even greater concern for future efforts to address gun violence.

Importantly, the symposium considers not simply the views of researchers, academics, and policymakers, but also the public. Thus, this symposium provides insight into the public’s views on gun safety and expansion of gun rights, as well as the extent to which policy priorities do or do not align with those views. In doing so, knowledge of gun violence disparities is examined as well as the potential for expansion of gun rights to exacerbate those disparities for the vulnerable, marginalized, and underserved. Finally, the symposium provides a look at paths forward that may garner more bipartisan support. By expanding our understanding of what qualifies as policies to address gun violence, emphasizing the importance of reducing suicide, and incorporating genuine stakeholders — those seeking to reduce gun violence without unnecessarily encroaching on gun rights — this symposium aims to provide guidance and some hope for the future.

Under *Bruen*, the constitutional inquiry consists of determining if the action is protected by the Second Amendment and, if so, whether historically there were sufficiently similar laws in enough jurisdictions to satisfy a majority of the justices.^17^ What qualifies as sufficiently analogous, how many jurisdictions are enough, and even what time periods are of most consequence remains unclear, leaving lower courts to guess when evaluating the deluge of legal challenges left in *Bruen*’s wake.^18^ But what is more transparent is that the Court has little concern for those currently impacted by gun violence.

Unlike other areas of constitutional law, including those related to our most protected fundamental rights, no longer does the analysis take into consideration the harm to others and the degree to which a law or regulation may mitigate that threat to the public. Thus, while the public and policymakers may be seeking a way to strike a balance between gun rights and gun safety the Supreme Court is limiting the ability of lower courts to do the same, generating significant uncertainty over the future of gun policies. Given the Court’s alleged interest in how their rulings impact public debate — a point raised in *Dobbs v. Jackson Women’s Health Organization* to overturn half a century of precedent^19^ — this binary approach focused solely on history will impede a balanced path forward that uses modern approaches to solve a contemporary problem.^20^ Whether *Bruen* ultimately undermines its own precedential force, as Justice Samuel Alito asserted for *Planned Parenthood v. Casey* in writing the *Dobbs* ruling,^21^ due to an inaccurate predictive judgment about providing a “more administrable” standard of review remains to be seen.^22^ But current trends in the lower courts are not promising.

This symposium endeavors to take stock of where we are as a country in light of the Supreme Court’s *Bruen* decision and our intractable political partisanship. While the Court and politicians appear content to frame the issue as gun rights *or* gun safety, these positions are not mutually exclusive. Importantly, the symposium considers not simply the views of researchers, academics, and policymakers, but also the public. This symposium provides insight into the public’s views on gun safety and expansion of gun rights, as well as the extent to which policy priorities do or do not align with those views. In doing so, knowledge of gun violence disparities is examined as well as the potential for expansion of gun rights to exacerbate those disparities for the vulnerable, marginalized, and underserved. Finally, the symposium provides a look at paths forward that may garner more bipartisan support. By expanding our understanding of what qualifies as policies to address gun violence, emphasizing the importance of reducing suicide, and incorporating genuine stakeholders — those seeking to reduce gun violence without unnecessarily encroaching on gun rights — this symposium aims to provide guidance and some hope for the future.

## I. Public Perceptions of Gun Rights and Gun Safety

United States gun culture is often cited as a significant contributor to the inability of the country to determine how to stem the growing tide of gun violence.. And while that almost certainly does play a role, the culture of partisanship and framing any politically sensitive issue as a win or lose binary may be just as relevant. Due to a system that does not produce representative governance — through laws that gerrymander political maps and restrict voting — political posturing has replaced the need to be responsive to public demand. For example, there is has been a consistent majority that has supported universal background checks for procuring firearms.^23^ Despite decades of consistent public backing, politicians in many states have responded to a much smaller fraction of financial contributors by spending their time, energy, and resources to draft, propose, and pass laws like Second Amendment sanctuaries that carry primarily rhetorical force on the campaign trail.

Even if the Supreme Court continues to limit the constitutional salience of empirical data on gun safety, this does not mean it cannot be used to inform and educate the public and policymakers. Many still believe that owning a firearm will make them safer, but data presented in this symposium by Ward and colleagues make it clear that a majority still believe the opposite to be true.^24^ According to their research, “most people do not agree that more wide-spread legal gun carrying will make them feel safer.”^25^ Therefore, the perception is not necessarily that firearms on a broad scale equates to increased safety or protection, but rather that people trust themselves to use firearms properly while having less faith in others to do the same. Even among gun owners, there is a fear of widespread guns in public.^26^ This research suggests there may be an opportunity to establish support not for gun bans or even gun control but gun safety. Even if gun owners believe that they know how to use their firearms safely, whether right or wrong, they may support policies that incentivize or even require people partake in safety measures such as training and receiving information on the benefits of safe storage. Such policies do not eliminate or even limit Second Amendment rights and, as a result, many may take important steps to reduce risk.

Meanwhile, the research conducted by Raissian and colleagues suggest political efforts to create “Second Amendment sanctuaries” are not doing so in response to citizen demand.^27^ Despite the alleged importance of Missouri’s Second Amendment Protection Act, Raissian et al. found that a majority of state residents had not even heard of the statute.^28^ According to their data, Missouri respondents tended to believe the law would increase murders, suicides, gun thefts, and would not reduce mass shootings.^29^ Even gun owners in the state did not report an expectation that the law would increase safety.^30^ As a result, the authors conclude that even in a state that may appear to have consensus on gun rights and the importance of minimal firearm regulations there is “a disconnect between policymaking and citizen engagement.”^31^

Given the current state of gun violence, it is disheartening to see elected representatives wasting time on symbolic laws that do not respond to citizen preferences, have little to no impact on improving gun safety or protecting gun rights, and simultaneously perpetuate partisan divide and undermine the rule of law. Instead of having the people driving the priorities and policies of government officials, we may see here evidence of the reverse — politicians and judges perpetuating the notion that there is a vast divide in public opinion with regard to gun violence, gun safety, and gun policies. As Raissian and coauthors suggest, questions should be raised to determine why deregulatory measures are pursued and prioritized when there is no evidence they will have a positive impact on public health or safety and they are not supported or expected to be beneficial by voters, including gun owners.^32^

## II. Expanding Gun Rights: Who Wins and Who Loses

One of the primary concerns with deregulation of firearms is that gun violence is not equitably distributed. As we think about the future of gun rights it is imperative to recognize that while Second Amendment doctrine increasingly frames the issue as one of personal responsibility, those most impacted are often unable to take personal action to protect themselves. Indeed, the populations disproportionately harmed by expansive Second Amendment interpretations are also more likely to be persecuted and prosecuted for attempts to exercise the very same principles underlying those rights.^33^ Self-defense — or legally justified self-defense to be more precise — is a matter of interpretation and, as a result, Second Amendment rights are subject to the realities of power and privilege. Thus, while marginalized groups are often used to justify the utility of expansive Second Amendment protections, they are more likely to be harmed by these efforts.

For example, Justice Clarence Thomas recounted the fear of newly freed slaves arming themselves after the Civil War, drawing a strong connection between armament and freedom.^34^ Setting aside the fact that what *Bruen* describes as a historically robust Second Amendment did not prevent the thousands of lynchings that took place after the Civil War, Justice Thomas refrained from discussing the current threat to Black communities, and Black men in particular, from gun violence. As compared to white Americans, Black Americans are ten times as likely to experience gun homicide, eighteen times as likely to suffer a gun assault injury, and almost three times as likely to be fatally shot by police.^35^ Black men account for 52% of gun homicides despite being less than 6% of the population.^36^ Racial disparities in gun violence have only worsened in recent years, with youth in non-white racial groups experiencing higher levels of exposure to neighborhood gun violence than white children during the COVID-19 pandemic, and Black children suffering the most.^37^ This helps to explain the result found by Ward et al. that there was “persistent safety concerns among many Black Americans, despite (in some cases) having personally acquired a gun.”^38^ The willingness to ignore racial disparities in gun violence is not going to help improve public awareness which, as Ward et al. demonstrate, is already lacking.^39^

But race was not the only demographic selectively used in *Bruen* to support expanding the Second Amendment right into the public sphere. In his concurrence, Justice Samuel Alito uses the example of a gay man in 1987 who was saved from would be attackers by a person carrying a concealed pistol.^40^ Yet, as Tobin-Tyler points out in her article LGBTQ+ people are twice as likely to experience gun violence as their cisgender and heterosexual counterparts.^41^ Justice Alito uses another example of a woman saved from an assailant by an armed bystander.^42^ But while the stranger danger provides a useful narrative for the value of carrying a firearm in public, women are much more likely to be victimized by someone they know, including a current or former male intimate partner.^43^ In fact, Tobin-Tyler’s discussion of two lower court cases striking down firearm restrictions for people under protective restraining orders illustrates the degree to which *Bruen* and its historical focus puts women at greater risk.^44^ So while firearm homicides related to intimate partner violence have increased 58% between 2014 and 2020, some lower courts said that *Bruen* bars this data from consideration and mandates that they examine laws from an era where marital rape was legal because married women were considered the property of their husband.^45^

What these carefully selected examples show is the difference between theory and reality. While self-defense may undergird the Second Amendment, the article by Light, Thomas, and Yakubovich details how race and gender infuence the perception of whether or not self-defense is justified.^46^ Since women are more likely to be victimized by men they know, they are at a distinct disadvantage when they defend themselves using firearms.^47^ Women’s use of firearms against their abusers has led to lengthy jail sentences, leaving abuse victims with difficult decisions especially taking into consideration the potential elimination of federal restrictions on firearms for those under restraining orders.^48^ The picture is even more troubling when using an intersectional lens, which Light, Thomas, and Yakubovich demonstrate is essential, with Black women 2.5 times more likely than white women to experience physical or sexual violence from a partner *and* less likely to seek help from law enforcement due to stigma and racism.^49^

Conversely, stand your ground laws, which allow the use of deadly force without any effort to retreat if an individual believes they are in danger, makes it subjectively justifiable to shoot strangers in public settings. But just as the harms of gun violence are not equitable, neither are these types of laws that allege to protect Second Amendment rights. Research on stand your ground laws shows that white shooters killing Black victims are five times more likely to be seen as justified than if the races are reversed.^50^ As a result Black people are both more likely to be victimized by gun violence and less likely to have their gun rights safeguarded under the law. The implicit biases of juries and judges — such as Black faces triggering thoughts of crime and the perception that Black males are larger and stronger than they actually are — influences who has the ability to exercise those Second Amendment rights for self-defense.^51^ This population-level data exhibits how the selective use of anecdotes conceals the realities of who is burdened by gun violence and put at greater risk from expansive Second Amendment interpretations.

## III. Moving Forward Through a Justice-Centered Firearms Framework

Instead of viewing *Bruen* as a death knell for opportunities to tackle gun violence, it may be worth considering it an opportunity to form a broader coalition pushing for policies that address the social determinants of health. Despite the suggestion that broad gun rights are a useful deterrent to crime and misuse of firearms, the primary drivers of criminal behaviors are social determinants such as poverty, neighborhood violence, and substandard housing.^52^ As Jay and Allen so pointedly state, “firearm carriage is as much a symptom as a cause of firearm violence,” especially for youth.^53^ To help stop the cycles of violence, or feedback loops, that they describe, those genuinely interested in reducing gun violence and protecting Second Amendment rights can find common ground.

Community violence intervention (CVI) programs, as well as place-based and structural interventions, can help reduce gun violence without any impact on gun rights. Importantly, CVI is distinguishable from enforcing criminal law and, as a result, reduces the reliance of and community interactions with law enforcement. Police shootings are gun violence, and the overpolicing of communities of color has done nothing to address the problem and instead has served to make it worse. By decreasing public trust and cooperation it has more likely contributed to reducing the probability of preventing and solving gun crimes. By contrast, CVI relies on credible frontline messengers that are part of the communities they seek to help.^54^ They rely on mentorship, address issues of trauma, and can provide training and job opportunities.^55^ In doing so, these programs have the potential to have an even larger impact beyond decreasing gun violence by reducing the mental and physical hardships that result from exposure to abusive policing tactics, as well as creating long-term skills for employment.^56^

Justifications for gun rights need not rely on the false notion that gun violence is an issue of personal responsibility or happens at random. Jay and Allen describe how racialized economic segregation is a strong predictor of gun violence, as redlining was not simply an issue of housing but also enabled public and private entities to disinvest in communities of color.^57^ Poverty drives crime and violence and, therefore, investments in areas suffering from higher levels of each can be an effective gun violence policy without ever implicating Second Amendment rights. For example, an anchor strategy is a place-based business approach that builds health and wealth in communities through local hiring, investing, purchasing, and community engagement.^58^ Rush University has undertaken an anchor program, making efforts to improve wealth building for their employees — many of whom live in West Side Chicago neighborhoods — by creating new career pathways, implementing pension reforms, and paying more than the regional living wage.^59^ Jay and Allen also describe how creating green spaces, improving abandoned houses, and eliminating unmaintained vacant properties qualify as “non-gun” gun violence policies as well.^60^

In their symposium article, Barnard and colleagues describe how community involvement can also be leveraged to tackle firearm suicides.^61^ Suicide is now the second leading cause of death among adolescents and young adults, with a 30% increase between 2000 and 2020.^62^ A majority of suicides are attributable to firearms, with that percentage increasing, and overall firearm suicides in 2021 reaching an all-time high since documentation began over three decades ago.^63^ The Barnard et al. article describes collaborative efforts in Colorado that incorporate the social-ecological model and stakeholders from the firearms community.^64^ Deploying similar methods to the CVI programs, community-based intervention suicide programs that rely on gun owners as trusted messengers are more likely to be successful in raising local awareness of firearm suicide and effective safety measures.^65^ In the program described by Barnard and coauthors, they use first responders and military leaders to raise awareness about the local firearm suicide problem while embedding information that could be used to reduce suicide risk.^66^

Conversely, some counties in Colorado response to an extreme risk protection order (ERPO) law passed in Colorado to combat suicides was to declare themselves Second Amendment sanctuaries.^67^ While these efforts do nothing to combat Colorado’s high suicide rates, which are fifth highest in the country, they do create unnecessary partisan tension over concern that the law will be used to unfairly remove firearms.^68^ Extreme risk protection orders (ERPOs) are a civil restraining order that allows for temporary removal of guns from someone who poses a threat to themselves or others.^69^ Thus, an ERPO could be used to legally remove firearms from someone who may be a risk to commit suicide. While programs to raise awareness of suicide and safety measures are vital, we cannot rely on these efforts alone. Public health history is replete with examples of limited success when relying solely on education and behavioral change. Data suggests that ERPOs have been successful in reducing suicides, and potentially mass shootings as well.^70^

Yet, in their article, Vitiello, Roskam, and Swanson share their concern that the Supreme Court may be limiting the protective potential of ERPOs.^71^ Relying primarily on law enforcement to determine when a firearm should be removed can create dangerous situations for both the citizen and police. Instead, Vitiello, Roskam, and Swanson urge for mental health providers to be more integrated into the process.^72^ As the authors explain in their article, “pathways for suicide are determined by many factors that interact in complex ways that are often unique to individual cases.”^73^ Law enforcement are not trained to evaluate these complexities and so their article contemplates how an expanded role for clinicians to be integrated into the process would be beneficial.^74^ In doing so, Vitiello, Roskam, and Swanson describe yet another scenario in which a judicial analysis of Fourth Amendment exceptions for exigent circumstances that is disconnected from reality will only put people at greater risk.^75^ Given that people can be involuntarily held — the most extreme form of restricting an individual’s rights and freedoms — under the very same justification of a potential threat to harm themselves or others, to strike down ERPOs would create a Second Amendment exceptionalism that places that right above the rights, interests, health, and safety of all others. Hopefully, this symposium helps to provide a better understanding of why that cannot, and should not, happen.

## IV. Conclusion

As demonstrated by research contained in this symposium, the future of protecting gun rights and tackling gun violence need not be a mutually exclusive choice. To be sure, the articles in this issue demonstrate quite clearly that much of the public feels the same. Therefore, while our courts and public officials continue to stoke flames of fear and put lives at risk, there is hope that a consensus can be created among those who truly believe a balance can be struck. But as we move forward with a coalition of those who believe in balance, it is imperative that we center the voices of those most effected by gun violence — communities of color, and the Black community in particular. Taking a page from the reproductive justice movement,^76^ a more justice-centered firearms framework is sorely needed.

Not all gun policies are an end to gun rights, but tackling gun violence is not merely about restricting gun rights. Moving forward, efforts to mitigate gun violence must include policies that increase green spaces, improve education, and address income inequality. Medicaid policy is gun violence policy. And for a justice-centered approach to incorporate the demands of the public, this means policies to reduce gerrymandering and improve voting rights are gun policies. This wide array of relevant policies makes plain that an interdisciplinary approach will be necessary. And the audience of the *Journal of Law, Medicine & Ethics* is a perfect group of experts to source this effort. My hope is that this symposium helps to inspire and inform those ready to join the fight.

## Note

The author has no conflicts to disclose.
